# Supplementary material for: MicroRNA-Mediated Responses to Cadmium Stress in Arabidopsis thaliana
Source: Plants (Basel). 2021 Jan 10;10(1):130. doi: 10.3390/plants10010130 (PMC7827075; doi:10.3390/plants10010130)
Supplement: Supplementary file 1 [file plants-10-00130-s001.pdf]

Supplementary Table S1: Oligonucleotide sequence of DNA primers used in this study. Primers listed were used for miRNA-specific cDNA synthesis, RT-qPCR assessment of mRNA transcript abundance or SL-RT-qPCR assessment of miRNA accumulation.

Transcript	Gene ID	Oligonucleotide sequence (5' to 3')
UBI10	AT4G05320	FP: GGCCTTGTATAATCCCTGATGAATAAG
		RP: AAAGAGATAACAGGAACGGAAACATA
SPL7	AT5G18830	FP: CACTGTACCTGTGACTGCGA
		RP: CACACAACAGCAACCTGACG
PDR8	AT1G59870	FP: TTATGGACGTTTTGGCCGGA
		RP: ACTGTTACTTGCGGGGAGTG
NRAMP6	AT1G15960	FP: TCCTCGGTGCCATGGTTATG
		RP: ATGCCGGTTACTGAACGAGG
FSD1	AT4G25100	FP: TCCATTCGCACTGGATGCTT
		RP: TGCTCTAAGGGCTTGCCTTC
HMAS2	AT4G30110	FP: GATAAGCATTGCAAGCCGGG
		RP: TCCGAACTGGCTTTAGCTGG
HMA4	AT2G19110	FP: AAACCCAAACAGCAGGTGGA
		RP: ATCTGCACCGGGACAACAAT
ARF10	AT2G28350	FP: TTTCCCGCGGCTTGATTACT
		RP: GGTTAGGAGATGACGGCGAG
ARF16	AT4G30080	FP: TTCGCCACCGAGGAAAAAGA
		RP: CCACGGGAACATTGTCCAGA
ARF17	AT1G77850	FP: TGCACATGCAACTCAACTGC
		RP: GACTTGGATCAGGTGCTGCT
ARF6	AT1G30330	FP: GTGTTCGTCGGTACATGGGT
		RP: CTTGACAGATCGCCAATGCG
ARF8	AT5G37020	FP: CCCAAGCCTACCACCACAAT
		RP: TCCTCCGGTGTCAATGGTTG
TIR1	AT3G62980	FP: GTGCTTGCTTCAACCTGCAA
		RP: TTTGGACAGCCCATGGAGAC
ATPS1	AT3G22890	FP: ATCTCCGGCACTAAGATGCG
		RP: ACCTGGGCACATAAAACCGT
PHO2	AT2G33770	FP: ACCGTTTCTCATCAAGGCGT
		RP: GTGCCCGTCCACCATAAGAA
LAC3	AT2G30210	FP: CCGTTCGACAACACAACCAC
		RP: GACTGGGAAAACAGGAGCGA
GRF1	AT2G22840	FP: CGTCGCATAAACAAGCCTCG
		RP: ATTTCAGCTCTTCGGGCCAA
GRF2	AT4G37740	FP: CTTGGCCTGAAGAGCTGACA
		RP: GTGTGTGGAGGAAGGGGATG
GRF3	AT2G36400	FP: CCATACGAGTCCCACATCGG
		RP: CTGAGCTCATGGGGCTTGAA
GRF7	AT5G53660	FP: CATCCCCCACCGTTAGATCG
		RP: TGCTTCCATGCTTCCGACAT
GRF8	AT4G24150	FP: GCTGCTGTGACTGTAGCAGA
		RP: CTCATGCCATTGAGCTTCGC

GRF9	AT2G42590	FP: CTCACATGAGAATGCCGGGT
		RP: ATCAGAAACTCGGGGCAGTG
Universal-SLR		RP: CCAGTGCAGGGTCCGAGGTA
snoR101		FP: CTTCACAGGTAAGTTCGCTTG
		RP: AGCATCAGCAGACCAGTAGTT
miR160		FP: AACGTGCCTGGCTCCCTGTATGCC
		RTSL: GTCGTATCCAGTGCAGGGTCCGAGGTATTCGCACTGGATACGACTGGCAT
miR167		FP: CGCGCCTGAAGCTGCCAGCATGATCTG
		RTSL: GTCGTATCCAGTGCAGGGTCCGAGGTATCCGCACTGGATACGACCAGATC
miR393		FP: CGGCGCCTCCAAAGGGATCGCATTGAT
		RTSL: GTCGTATCCAGTGCAGGGTCCGAGGTATTCGCACTGGATACGACGATCAA
miR395		FP: GCGCTGAAGTGTTTGGGGGAACTC
		RTSL: GTCGTATCCAGTGCAGGGTCCGAGGTATTCGCACTGGATACGACGAGTTC
miR396		FP: GCGCGTTCCACAGCTTTCTTGAAC
		RTSL: GTCGTATCCAGTGCAGGGTCCGAGGTATTCGCACTGGATACGACAAGTTC
miR399		FP: GCATGCCAAAGGAGATTTGCCCTG
		RTSL: GTCGTATCCAGTGCAGGGTCCGAGGTATTCGCACTGGATACGACCAGGGC
miR408		FP: ACGACAGGGAACAAGCAGAGCATG
		RTSL: GTCGTATCCAGTGCAGGGTCCGAGGTATTCGCACTGGATACGACCATGCT
